# Uropygial secretion changes mouth colouration in starling nestlings

**DOI:** 10.1098/rsbl.2024.0535

**Published:** 2024-11-20

**Authors:** Antonio José García-Núñez, Juan José Soler, Ester Martínez-Renau, Gustavo Tomás

**Affiliations:** ^1^ Departamento de Ecología Funcional y Evolutiva, Estación Experimental de Zonas Áridas (CSIC), Almería 04120, Spain; ^2^ Unidad Asociada (CSIC): Coevolución: Cucos, Hospedadores y Bacterias Simbiontes, Universidad de Granada, Granada 18071, Spain

**Keywords:** colouration, mouth flanges, makeup, parent–offspring communication, reflectance, uropygial secretion

## Abstract

Cosmetic colourations of animals have been mainly studied in scenarios of sexual selection, while there has been no assessment of the partial contribution of cosmetics to the final colouration of begging-related traits. In birds, the uropygial gland is functional soon after hatching, and we experimentally investigated the effects of uropygial secretion on mouth-flange colouration in spotless starling (*Sturnus unicolor*) nestlings. Nestlings' flange colouration was measured with a spectrophotometer before and after being either cleaned of, or painted with, its own uropygial secretion. After cleaning, flanges were brighter, more UV and less yellow-red coloured (chroma). Instead, painting the mouth flanges of nestlings with their own uropygial secretion did not modify any of the considered colour components. Our findings therefore show that the uropygial secretion of nestlings alters their beak colouration and open the possibility for future research to investigate the role of uropygial secretion in reinforcing the signalling role of begging-related traits involved in parent–offspring communication.

## 1. Introduction

Besides chemical colourations arising from the accumulation of pigments and physical colourations resulting from nanostructures, a third factor that could influence the colouration of a particular trait is the cosmetic application of natural products [1]. Cosmetics, which can be obtained from the environment (e.g. from soil) or can be produced by the animal itself (e.g. gland and skin secretions), could have a role in animal communication [2–4]. For instance, when bearded vultures (*Gypaetus barbatus*) change their plumage with soil stained with iron oxides, they are likely showing social status to conspecifics and reduce costly agonistic encounters [2]. Similarly, when hoopoe females stain the eggshell with uropygial secretion, they show the antibiotic quality of their uropygial secretion to their partners, who respond by increasing their feeding effort [5].

The scant research on cosmetic colouration related to uropygial secretion in birds has primarily concentrated on contexts associated with sexual selection [4], while its potential role in parent–offspring communication has even more rarely been considered [6,7]. The uropygial (or preen) gland of birds produces a blend of waxes, fatty acids and hydrocarbons [8] with colourations that differ among species [9]. Thus, when the uropygial secretion stains particular integuments, it modifies the spectral characteristics of reflected light by selectively absorbing or reflecting it within specific wavelength ranges [9]. In nestling birds, the uropygial gland starts producing secretion soon after hatching (e.g. in passerines, the second or third day [8, p. 249]) and, thus, it may reach their integuments involved in parent–offspring communication along the nestling period. Moreover, yellow-red [10,11] and UV- [12] rich colourations of nestlings typically associate with their oxidative and immunological condition and influence parental feeding decisions [7,13–16]. Then, if the uropygial secretion reaches those coloured traits of nestling birds, it would likely modify their colouration and influence parent–offspring communication, at least partially.

In the present study, the flanges of spotless starling (*Sturnus unicolor*) nestlings were experimentally cleaned of, or smeared with, uropygial secretion and the change in colouration was assessed. We focussed on colour components that have been previously shown to predict body condition and parental feeding favouritism in this species (i.e. brightness, UV and yellow-red chromas) [7,11]. We predict that significant changes in colour components of flanges should occur after cleaning them of natural remains and uropygial gland secretion. Instead, if uropygial secretion naturally stains the nestlings’ mouth flanges, experimental smearing with their own uropygial secretion should result in relatively smaller or subtle colour changes.

## 2. Material and methods

Fieldwork was conducted from April to June, during the breeding season of 2024 in a population of spotless starlings (hereafter starlings) breeding in nest-boxes in the old rail station of La Calahorra, southern Spain (37°15′ N, 3°01′ W). Regular monitoring of nest-boxes allowed determination of hatching dates. Eleven days after hatching, we measured the natural colouration of the right flange of the two heaviest nestlings per nest, and each one was randomly assigned to a different experimental treatment (i.e. pairwise experimental design). The first treatment involved cleaning the same flange with a cotton swab (cleaned), while the second treatment involved smearing nestling flange with its own uropygial secretion, extracted with the aid of a microcapillary tube and immediately used to smear the flange (painted). After the experimental treatment, we obtained a second measure of flange colouration.

We measured colouration with an Ocean Optics S2000 spectrophotometer, estimating the overall brightness across the entire wavelength range (300–700 nm), and chroma (i.e. percentage of reflectance) within two specific intervals (UV: 300–400 nm, yellow-red: 580–680 nm) (see [17]). We recorded three colour measures, which were later averaged, for both before and after the treatment of flanges, following established protocols [11,17]. To process the data, we applied AVICOL v. 6 [18] to set all negative reflectance values to zero and reduce noise using a triangular correction integrated into the software.

### (a) Data analyses

We used repeated-measures multivariate analysis of variance (MANOVA) to estimate separately the effects of the experimental cleaning or painting the nestlings’ mouth flanges on colouration, and reported Wilks’ lambda and associated *p*-values as general effects and univariate results for each analysed colour component. All variables used in the MANOVA analyses were previously tested for homoscedasticity, and they met this assumption. Experimental effects on different colour variables (i.e. univariate results) are also shown. The experiments were performed in first and second breeding attempts that might differ in resource availability and, thus, affect colouration of signals of phenotypic condition [19]. However, after controlling for the effect of body mass, flanges colouration did not depend on the breeding attempt (general linear model, *p* > 0.40) and, then, it was not considered further in the analyses. Statistical analyses were run in Statistica v. 13.0 [20].

## 3. Results

**Figure 1.**
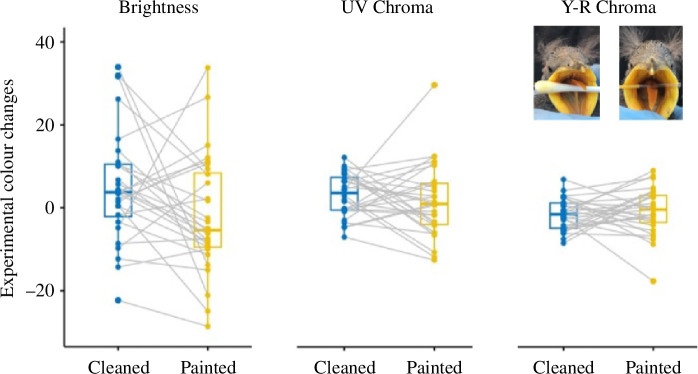
Flange colour changes (brightness, UV chroma, yellow-red (Y-R) chroma) of spotless starling nestlings before and after experimental cleaning of or painting with uropygial secretion. Lines connect siblings of the same nest that were the subject of different experimental treatments. Box plots show the median (horizontal line), lower quartile (median to end of box), upper quartile (median to top of box), minimums and maximums within 1.5× interquartile range (whiskers), and outliers (data points beyond the whiskers). Pictures show a cotton swab that was used to clean flanges of the nestling (left) and a capillary containing yellow uropygial secretion that was used to paint nestling flanges.

Experimental cleaning of flanges of starling nestlings influenced colouration (Wilks = 0.51, *F* = 8.32, d.f. = 3,26, *p* = 0.0005). It increased brightness (*F*_1,28_ = 5.14, *p* = 0.031) and UV chroma (*F*_1,28_ = 11.06, *p* = 0.002), while yellow-red chroma decreased (*F*_1,28_ = 4.99, *p* = 0.033) (figure 1). Instead, painting flanges did not significantly affect colouration (Wilks = 0.85, *F* = 1.59, d.f. = 3,26, *p* = 0.215). However, although none of the measured colour variables significantly varied after the experiment (brightness: *F*_1,28_ = 0.03, *p* = 0.866; UV chroma, *F*_1,28_ = 1.16, *p* = 0.289; and yellow-red chroma: *F*_1,28_ = 0.58, *p* = 0.452), the non-significant effects were in the same direction than those produced by the cleaning experiment (figure 1). In accordance, the interaction between experimental treatments (painting or cleaning) and its effects (repeated measures) did not reach statistical significance (Wilks = 0.95, *F* = 0.99, d.f. = 3,54, *p* = 0.404, colour variables, *F* >1.45, d.f. = 1,56, *p* > 0.23).

## 4. Discussion

Our main findings are that removing uropygial secretion from the mouth flanges of spotless starling nestlings but not painting them with their own secretion modifies their colouration. Below we discuss the importance of these results supporting the idea that uropygial secretion serves as a natural cosmetic agent with a possible signalling function.

Mouth flanges of spotless starling nestlings, as well as their uropygial secretion, are yellow coloured to the human eye with a peak at the UV wavelength [7]. Moreover, brightness, UV and yellow-red chroma of the flanges are associated positively with body condition and/or plasma carotenoid concentration [11], while UV and yellow-red chroma of nestlings’ mouth are associated positively with parental feeding preferences [7]. This previous knowledge therefore suggests a role of these colour components in parent–offspring communication. Finally, cotton swabs turned to yellow colour after rubbing nestling flanges with them, which suggest that the colour-changing effects of the uropygial secretion determine the colouration of nestling flanges, at least partially [7]. The experimental cleaning of nestlings’ mouth flanges increased their brightness and UV chroma but decreased yellow-red chroma. Those effects could therefore generate contradictory information for parents. Achromatic and UV colourations of cleaned mouth flanges would indicate to parents that nestlings are of better phenotypic quality [14], while their modified yellow-red colour would suggest to adults that their phenotypic condition is worse. Thus, since starling adults tended to preferentially feed nestlings with mouth colour reflecting better conditions, inferring the net effect of colour differences between natural and cleaned nestlings’ mouth is not straightforward and needs further investigation.

The detected effects of cleaning mouth flanges could however be explained by the dust and food remains that, together with the uropygial secretion, were removed after cotton swabbing. Nevertheless, painting mouth flanges of nestlings with their own uropygial secretion did not significantly modify their colouration in terms of brightness, UV or yellow-red chromas, which further confirms that the natural colour of nestlings’ mouth included the effect of stained uropygial secretion. The non-significant effect of painting mouth flanges was in the same direction as the detected effect of cleaning, which may be due to partial removal of dust and food remains when painting mouth flanges. Independently of the reasons explaining the direction of the experimental effects, experimental manipulations, such as impeding the use of uropygial secretion by nestlings, would help to determine whether the effect of uropygial secretion on nestling colouration is a signal to parents or a non-adaptive by-product of nest occupancy, with the oil having other functions.

The colour-changing effects of uropygial secretion that we here demonstrated in spotless starling nestlings might be widespread within the avian phylogeny. Uropygial secretion is not completely transparent, and, as we demonstrated in starlings, it may have a dual impact on the colouration of the body surfaces where it is applied, increasing or decreasing achromatic brightness and modifying the spectral characteristics of reflected light by selectively absorbing or reflecting it in the entire or within specific wavelength ranges [6,9,21,22]. Then, it is likely that the effects of the secretions on nestlings affect the colour of begging-related traits and therefore parental provisioning effort and food allocation decisions [15]. Uropygial secretion might, however, oxidize or disappear quickly, and, in the absence of frequent preening behaviour, the colour of smeared body parts may change over time [4,23]. Consequently, frequent preening activity would be required to maintain the colouration of particular integuments. Both preening activity and production of coloured secretion are likely costly and, thus, the relatively frequent use of secretion as a cosmetic could contribute to maintain the honesty of the colour signals within the flamboyant mouth of nestling birds, a possibility that should be further investigated. In conclusion, our results support the idea that smeared uropygial secretion modifies the colouration of nestling flanges. Future research should focus on determining the impact of secretion on gape colour in terms of associated costs and differential parental favouritism.

## Data Availability

Raw data and a description of the data can be found openly and permanently available at [24].
